# *Mycoplasma bovis* co-infection with bovine viral diarrhea virus in bovine macrophages

**DOI:** 10.1186/s13567-017-0499-1

**Published:** 2018-01-09

**Authors:** Nina Bürgi, Christoph Josi, Sibylle Bürki, Matthias Schweizer, Paola Pilo

**Affiliations:** 1 Institute of Veterinary Bacteriology, Department of Infectious Diseases and Pathobiology, Vetsuisse Faculty, University of Bern, Laenggass-Str. 122, 3001 Bern, Switzerland; 2 Graduate School for Cellular and Biomedical Sciences, University of Bern, Bern, Switzerland; 3 Institute of Virology and Immunology, Federal Food Safety and Veterinary Office (FSVO) and Department of Infectious Diseases and Pathobiology, Vetsuisse Faculty, University of Bern, Laenggass-Str. 122, 3001 Bern, Switzerland

## Abstract

Several studies suggest that synergisms between *Mycoplasma bovis* and other microorganisms might exacerbate disease outcome of bovine mycoplasmosis. Screening several bovine cell types to assess their potential use as in vitro infection models for *M. bovis*, it was observed that a widely used cell line of bovine macrophages (Bomac cells) is in fact persistently infected with bovine viral diarrhea virus (BVDV). The cell line was first cured of this virus allowing comparative studies between both cell lines. Subsequently, uptake and co-culture of two *M. bovis* strains of different clonal complexes with Bomac cells contaminated with BVDV and in BVDV-free Bomac cells were assessed. Additionally, cell viability, cytotoxicity and induction of apoptosis after infection with *M. bovis* were evaluated. No differences in the levels of uptake and growth in co-culture were observed between the two Bomac cell types and both *M. bovis* strains. Cytotoxicity was increased after infection of BVDV-free cells with one of the two strains, while apoptotic cell death was slightly induced by this strain in both cell lines. Overall, the presence or absence of BVDV in Bomac cells did not grossly change the parameters tested upon infection with *M. bovis*. Nevertheless, this cell model is very useful when studying viral co-infections with bacteria and could also be used for multiple co-infections. Considering the broad contamination of cell cultures with BVDV, careful screening for this virus should routinely be performed as its presence might be relevant depending on the molecular mechanisms being investigated.

**Electronic supplementary material:**

The online version of this article (10.1186/s13567-017-0499-1) contains supplementary material, which is available to authorized users.

## Introduction

*Mycoplasma bovis* is one of the major causative agents of bovine mycoplasmosis [1]. This disease has a broad range of clinical manifestations including pneumonia, mastitis, polyarthritis, otitis media and genital disorders in cattle [2–5]. In vivo antibiotic treatments are inefficient and no effective commercial vaccine is available [6]. Although this bacterium was first isolated in 1961 [7], the molecular mechanisms involved in the pathogenesis of bovine mycoplasmosis due to *M. bovis* are still poorly understood. Several studies suggest a multifactorial origin for disease development [1]. Thus, variable surface lipoproteins [8–13], adhesion and uptake by host cells [14–20], modulation of the host’s immune system [21–26], biofilm formation [27], synergistic interactions with other bacterial or viral pathogens [28–31] and the release of secondary metabolites [32, 33] were investigated. An interesting aspect observed in previous experimental studies and by analyzing material collected from natural infections is the synergistic interaction of *M. bovis* with other bacterial or viral pathogens in the development of severe lesions [28, 29]. The main microorganisms suspected to play a role in this process are *Pasteurella multocida*, *Mannheimia haemolytica*, *Histophilus somni*, bovine respiratory syncytial virus (BRSV), bovine herpes virus 1 (BHV-1), bovine viral diarrhea virus (BVDV) and parainfluenza virus type 3 (PIV-3), although some studies showed contradictory findings [1, 31, 34]. For this reason, in vivo and in vitro experimental data focusing on co-infections and host response are necessary to dissect the mechanisms involved in these potential synergistic interactions. Intracellular localization of *M. bovis* in host cells, including macrophages, was previously shown in vivo, but in vitro data were missing until recently [18–20, 35]. Lately, two research groups demonstrated in vitro uptake of *M. bovis* by several primary bovine cell types, including peripheral blood mononuclear cells (PBMCs), erythrocytes and turbinate cells [21, 36, 37]. However, the drastic cytotoxic effect of *M. bovis* on bovine endothelial cells, PBMCs and alveolar macrophages was not observed [37, 38]. Moreover, the induction of apoptosis following cell infection with *M. bovis* has been rarely studied, with inconsistent results observed with PBMCs and epithelial cells [21, 23, 39], and even a delay in apoptosis with bovine peripheral monocytes [22]. In addition, the synergistic effects of co-infections on cell uptake of *M. bovis*, cytotoxicity towards cells and induction of apoptosis have not been studied in vitro to date. Thus, the gathering of experimental data while testing standardized cell models is fundamental when investigating these topics in more detail and to understand the mechanisms involved. Accordingly, in vitro cell models are indispensable when dissecting the molecular and cellular details of biological processes occurring in specific cell types [40]. A point to ponder is the choice between primary cells and/or cell lines. Both systems are useful and have advantages and drawbacks [41]. Primary cells mimic the in vivo situation better than cell lines but primary cells originate from several individual animals and are thus prone to exhibit high variability between experiments and between different laboratories. Additionally, the isolation of primary cells is time consuming and these cells are in general less characterized. On the other hand, cell lines are “immortalized” and are in general well-characterized, very convenient to work with, show less variation between experiments and, importantly, are amenable to genetic engineering [42], which makes them first choice for initial studies aiming to characterize specific cell features.

In the present study, a bovine macrophage (Bomac) cell line, developed from bovine peritoneal macrophages by Stabel and Stabel in 1995 [43], was tested as an infection model for *M. bovis* to further investigate cellular mechanisms involved in mycoplasma–Bomac cell interaction. This cell line is widely used in research but proved to be contaminated with BVDV. The cell line was cured of the virus, and both BVDV-infected and BVDV-free Bomac cells were tested for mycoplasmal uptake, growth in co-culture, viability, cytotoxicity and induction of apoptosis after infection with *M. bovis*. The aim of this study was to compare the parameters mentioned above in both BVDV-infected and BVDV-free Bomac cells.

## Materials and methods

### Bacterial strains and axenic growth conditions

*Mycoplasma bovis* strain JF4278, isolated from the milk of a cow with mastitis and pneumonia in Switzerland in 2008 [44] and *M. bovis* strain L22/93, isolated from the lung of a cow in Switzerland in 1993, were filter-cloned and used for the experiments. These two strains were chosen as representative strains of the two distinct clonal complexes (CC) isolated in Switzerland. Strain JF4278, which belongs to the currently circulating clonal complex CC1, is associated with an increase of reported cases of severe mastitis. Strain L22/93 belongs to CC5 and was circulating in Switzerland until around 2007 [45]. The strains were pre-cultured for 20 h in SP4 broth medium [46] supplemented with 50 μg/mL cefoxitin sodium salt (Sigma-Aldrich, Buchs, Switzerland) or for 4–5 days on agar plates at 37 °C in a humidified atmosphere. The concentration of mycoplasmas of all liquid pre-cultures was measured by performing 10-fold serial dilutions and plating on SP4 agar plates. A growth curve of both strains in SP4 broth medium was performed. Ten microliter of the mycoplasmas frozen stock was added to 990 μL of SP4 broth medium and incubated for 20 h to have approximately 6 × 10^8^ cells/mL (strain JF4278) or 1 × 10^8^ CFU/mL (strain L22/93). 10-fold serial dilutions were further performed in a volume of 2 mL SP4 broth medium and all dilutions were plated on SP4 agar plates to confirm the concentration of mycoplasmas of the pre-culture. To measure growth curves starting with approximately 100 CFU/mL, the dilution 10^−7^ (strain JF4278) and the dilution 10^−6^ (strain L22/93) were used as starting cultures (time point 0). One hundred μL of the culture were taken at time points (hours): 0, 6, 24, 30, 48, 54 and 72. Ten microliter of 10-fold serial dilutions were plated on SP4 agar plates and incubated. Subsequently, colonies were counted under a stereomicroscope. The assay was performed in three independent experiments.

### Bacterial generation time in axenic medium

The generation time of both strains of *M. bovis* during the exponential phase was calculated using the data obtained for axenic growth with the following formula [47]:

$${\text{G}} = \frac{\text{t}}{\text{n}}$$

*G* represents the generation time in hours, *t* the interval of time and *n* the number of generations. The number of generation *n* is first determined with the formula described below:

$${\text{n}} = \frac{{\log \left( {\text{a}} \right) - { \log }({\text{A}})}}{{{ \log }(2)}}$$

*a* represents the number of bacteria at the end of the time interval, while *A* is the number of bacteria at the beginning of a time interval. *A* is the number of bacteria at time point 6 h and *a* is the number of bacteria at 30 h.

### Bovine macrophage (Bomac) cell lines

Bomac cells [43, 48] were maintained at 37 °C in a humidified 5% CO_2_ atmosphere in minimal essential medium (MEM)-Earle medium supplemented with 2.2 g/L NaHCO_3_ (Biochrom, Berlin, Germany), 1% l-glutamine (Biochrom), 7% fetal calf serum (Gibco Life Technologies, Rockville, USA) and 1 × penicillin–streptomycin mixture (Gibco). Cells were routinely screened to ensure the absence of contamination with mycoplasmas using PCR with the Venor^®^GeM kit (Minerva Biolabs, Berlin, Germany) and BVDV using immunostaining with an in-house swine anti-BVDV hyperimmune serum (National Reference Center for BVDV, Institute of Virology and Immunology) as previously described [36]. The original Bomac cell line present in the laboratory [48] was found to be positive for BVDV. Subtyping of the virus [49] revealed the presence of BVDV genotype-2. The cell line was subsequently cured of the BVDV by several passages in the presence of the aromatic cationic compound DB772 [50], a known inhibitor of pestivirus replication (Marti and Schweizer, manuscript in preparation). This second cell line, i.e., Bomac cured of BVDV, was maintained and grown for experiments using the same conditions as the cell line infected with BVDV. Approximately 4 × 10^4^ bovine cells were seeded per well containing 0.5 mL of medium in 24-well plates (TPP^®^) for cell infections and gentamicin protection assay and 8 × 10^3^ bovine cells per well containing 100 μL in 96-well plates (Greiner Bio-One, Frickenhausen, Germany) for the ApoTox-Glo™ Triplex Assay in MEM-Earle without antibiotics 1 day before the experiments. The lowest passage used was 23 for Bomac cells free of BVDV and 31 for Bomac cells infected with BVDV. The cells were used for the following 10–40 passages until the end of the experiment without detection of overt changes during passaging.

### Cell infection and gentamicin protection assay

The cell infection and gentamicin assays were adapted from a previous study using primary embryonic calf turbinate (PECT) cells [36]. The two strains of *M. bovis* were grown for 20 h as described above, then 1.5 mL of the pre-cultures were centrifuged 15 min at 5900 × *g* and washed once in PBS at pH 7.5. Mycoplasmas were further suspended and diluted in MEM-Earle medium to infect Bomac cells at a multiplicity of infection (MOI) ranging between 2 and 10 (Additional file 1). The MOI is defined as the number of added bacteria per individual host cell. The plate was centrifuged for 5 min at 600 ×* g* to synchronize infection. After 3 h of infection, cells were washed twice with PBS followed by the addition of fresh MEM-Earle medium (cell infection assay) or MEM-Earle medium supplemented with 400 μg/mL gentamicin sulfate (TOKU-E Company, Bellington, USA) (gentamicin protection assay). Plates were incubated for 3 additional hours (total of 6 h post-infection). Cells were subsequently washed three times with PBS to remove gentamicin and fresh MEM-Earle medium was added to each well. To evaluate uptake of mycoplasmas and survival, sampling was performed at three time points, i.e., at 0, 6 and 54 h post-infection. For the collection of samples at all time points, wells were washed with PBS and bovine cells were scraped and lysed mechanically using a 23-gauge needle and a syringe. Samples were taken to determine the CFU/well by performing 10-fold serial dilutions in PBS and plating the mycoplasmas on SP4 agar plates. As controls, assays were performed with the two *M. bovis* strains without Bomac cells to ensure efficient killing of extracellular mycoplasmas by gentamicin and were performed for each experiment with each cell line. These controls were performed in Eppendorf tubes and before washings; bacteria were centrifuged for 15 min at 5900 × *g*. Additionally, the survival of *M. bovis* strains JF4278 and L22/93 in MEM-Earle medium and in MEM-Earle preincubated with both Bomac cell lines cells for 24 or 48 h was assessed as previously described [36]. The assays were performed in triplicates in three independent experiments.

### ApoTox-Glo™ Triplex Assay

The ApoTox-Glo™ Triplex Assay (Promega, Madison, USA) assesses viability and cytotoxicity by a combination of cell-permeant and non-permeant protease substrates, detecting changes in cell membrane integrity and caspase-3/7 activation, a hallmark for apoptosis induction. Briefly, cell viability is measured using a cell permeant substrate of a host protease that is inactivated when released in the cell culture medium. Cytotoxicity is quantified by the cleavage of a substrate that is unable to pass through membranes but is cleaved when the protease is released in the cell culture medium. For apoptosis, a luminogenic substrate, is cleaved by activated caspase-3/7. Preliminary tests were performed to evaluate the induction of apoptosis in Bomac cells after treatment with staurosporine (Abcam plc, Cambridge, UK). Staurosporine was tested at final concentrations of 0, 0.5, 2.5, 5 and 15 µM in MEM-Earle medium (Additional file 2). Twenty-four hours prior to the experiment, Bomac cells were seeded in black 96-well plates with a clear bottom (Greiner Bio-One). Subsequently, cell medium was replaced with fresh medium. For the staurosporine tests, the different concentrations of staurosporine were added to the cells 18 h after the addition of fresh medium and were incubated for an extra 6 h before performing the measurements. The ApoTox-Glo™ Triplex Assay was performed according to the manufacturer’s protocol. Fluorescence and luminescence signals were read with a Cytation 5 Cell Imaging Multi-Mode Reader (BioTek Instruments GmbH, Luzern, Switzerland) with the filter settings suggested in the ApoTox-Glo™ Triplex Assay protocol. For subsequent experiments, 2.5 µM staurosporine was chosen since this concentration represents the beginning of the plateau of apoptosis induction in both cell lines used in this study.

To perform the ApoTox-Glo™ Triplex Assay with mycoplasmas, Bomac cells were seeded as described above. The cell medium was changed 24 h after cell seeding, and Bomac cells were infected with *M. bovis* strains JF4278 and L22/93 at an MOI of approximately 5. At 18 h post-infection, 2.5 µM staurosporine was added to selected samples for an additional 6 h. After a total of 24 h of infection with *M. bovis* the ApoTox-Glo™ Triplex Assay was performed according to the manufacturer’s protocol. Fluorescence and luminescence signals were read with a Cytation 5 Cell Imaging Multi-Mode Reader as described above. All experiments were performed three times in duplicates.

### Statistical analysis

*Mycoplasma bovis* titers (log(10) [CFU/well]) measured during infection assays are shown as means ± standard deviations of mean values from three independent experiments. The significance of differences between individual groups and strains in the same group in the gentamicin assay was calculated with the Welch’s *t*-test. For the ApoTox-Glo™ Triplex Assay, fluorescence- and luminescence-units for viability, cytotoxicity and apoptosis signals were displayed as relative values to uninfected cells. The significance of differences between uninfected and infected cells with either strain was calculated with the Welch’s t-test. Statistical analysis was performed using the software GraphPad Instat™ V2.05 (GraphPad Software Inc., La Jolla, CA, USA).

## Results

### *M. bovis* strain JF4278 reaches higher concentrations in SP4 medium and has a lower generation time than strain L22/93

**Figure 1:**
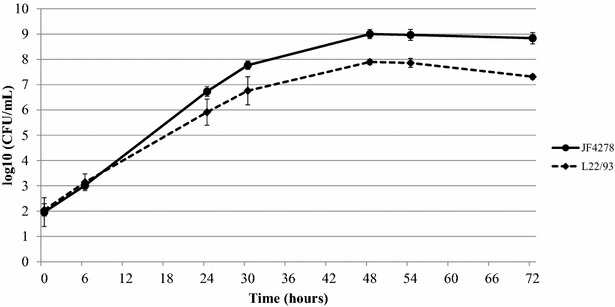
**Growth curves of** ***M. bovis*** **in axenic medium.** The straight line represents strain JF4278, while the dashed line represents strain L22/93. The x-axis indicates the timepoints and the y-axis the log10 CFU/mL. The data shown are the mean values of three independent experiments. Standard deviations of individual measurements per time point are indicated as vertical bars.

The growth of *M. bovis* strains JF4278 and L22/93 in axenic medium (SP4) was measured. As shown in Figure 1, strain JF4278 reaches a concentration of one log10 more than strain L22/93 in SP4 medium before attaining the plateau of stationary phase. Additionally, after 54 h of growth, the strain L22/93 enters the death phase (Figure 1). The generation time of both *M. bovis* strains in axenic medium (SP4) was calculated. The generation time of strain L22/93 was higher compared to JF4278. JF4278 had a generation time of 1.52 h ± 0.08, while L22/93 had a generation time of 2.01 h ± 0.16.

### *M. bovis* is internalized and grows in co-culture with BVDV-infected and cured Bomac cell lines

**Figure 2:**
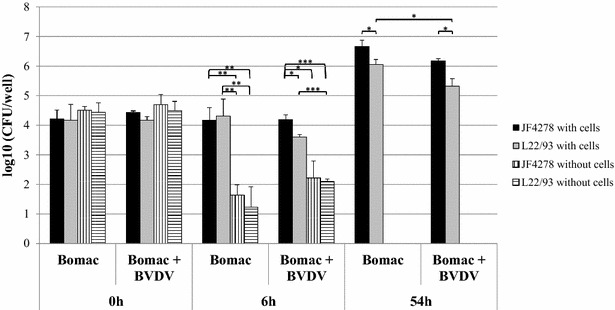
**Infection model of** ***M. bovis*** **in Bomac cells.** Time points 0, 6 and 54 h post-infection with an MOI between 2 and 10 are shown. Black columns correspond to strain JF4278 (co-culture), while grey columns correspond to strain L22/93 (co-culture). Vertically striped columns correspond to strain JF4278 alone in cell medium, while horizontally striped columns correspond to strain L22/93 alone in cell medium. The x-axis represents the number of hours post-infection (Bomac: Bomac cells free of BVDV; Bomac + BVDV: Bomac infected with BVDV), while y-axis represents the log10 CFU/well. The data shown are the mean values of triplicates of three independent experiments. Standard deviations of individual measurements are indicated as vertical bars. *P < 0.05, **P < 0.01, ***P < 0.001.

**Figure 3:**
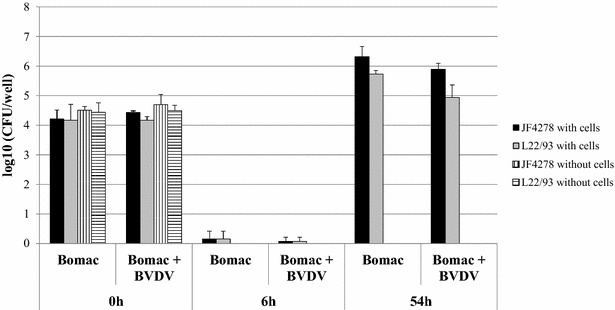
**Gentamicin protection assay.** Time points 0, 6 and 54 h post-infection with an MOI between 2 and 10 are shown. Gentamicin was added 3 h post-infection and time 6 h post-infection corresponds to the end of the gentamicin treatment. Black columns correspond to strain JF4278 (co-culture), while grey columns correspond to strain L22/93 (co-culture). Vertically striped columns correspond to strain JF4278 alone in growth medium, while horizontally striped columns correspond to strain L22/93 alone in growth medium. The x-axis represents the number of hours post-infection (Bomac: Bomac cells free of BVDV; Bomac + BVDV: Bomac infected with BVDV), while y-axis represents the log10 CFU/well. The data shown are the mean values of triplicates of three independent experiments. Standard deviations of individual measurements are indicated as bars. *P < 0.05, **P < 0.01, ***P < 0.001.

In order to evaluate mycoplasmas uptake and growth in co-culture of *M. bovis* with Bomac cells, cell infections and gentamicin protection assays were performed with Bomac cells as previously described for PECT cells [36]. For each experiment, controls including *M. bovis* in cell culture medium without addition of gentamicin with and without Bomac cells were included. The incapacity of *M. bovis* to multiply in MEM-Earle medium was confirmed (Additional files 3 and 4). However, growth in spent MEM-Earle medium, from both cell lines, was observed (Additional files 3 and 4). Six hours post-infection without gentamicin treatment and in co-culture with both cell lines, no significant differences between both *M. bovis* strains were noticed apart from a faint decrease in recovered L22/93 when co-infected with BVDV (Figure 2). Furthermore, a limited number of *M. bovis* cells were persisting in the cell medium when Bomac cells were not present. Fifty-four hours post-infection, fewer bacteria of the strain L22/93 were recovered in BVDV-infected than in BVDV-free Bomac cells, whereas the difference for the strain JF4278 was not significant (Figure 2). No more viable bacteria were detected 54 h after the inoculation of *M. bovis* in medium without eukaryotic cells (Figure 2). After the gentamicin treatment (6 h post-infection), no viable mycoplasmas were detected without co-cultivation with Bomac cells (Figure 3). After 6 h of co-cultivation with Bomac cells, no significant differences in the number of viable bacteria were observed between the two strains tested and between Bomac cells infected with BVDV and Bomac cells free of BVDV (Figure 3). At 54 h post-infection, a slight decrease in the number of viable L22/93 compared to JF4278 and a small reduction of each strain in BVDV-infected versus non-infected cells was noticed but without statistical significance (Figure 3).

### *M. bovis* strain JF4278 induces slight cytotoxicity and apoptosis

**Figure 4:**
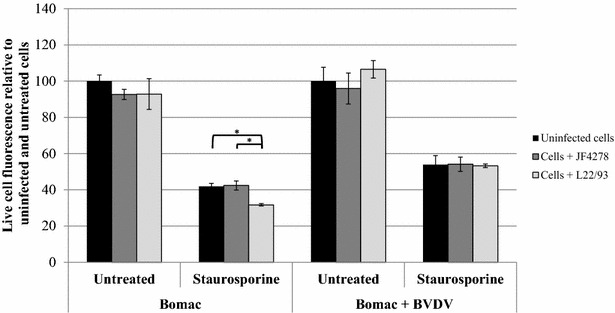
**Viability assay.** Time point 24 h post-infection with an MOI of approximately 5 is shown. Black columns correspond to uninfected cells, dark grey columns correspond to strain JF4278 (co-culture), while light grey columns correspond to strain L22/93 (co-culture). The data shown are the mean values of duplicates of three independent experiments. The x-axis represents the conditions tested (Bomac: Bomac cells free of BVDV; Bomac + BVDV: Bomac infected with BVDV), while y-axis represents the values of the measured test relative to untreated and uninfected cells. The values obtained for Bomac cell lines infected with *M. bovis* were normalized to values of the corresponding uninfected and untreated cells of each cell line. Standard deviations of individual measurements are indicated as bars. *P < 0.05, **P < 0.01, ***P < 0.001.

**Figure 5:**
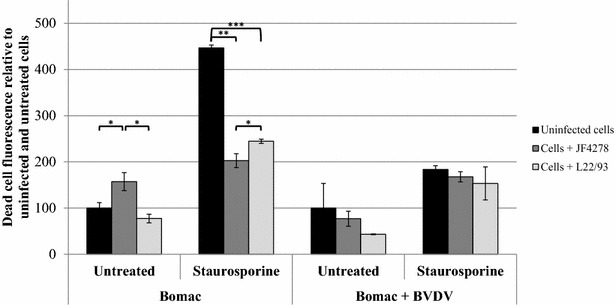
**Cytotoxicity assay.** Time point 24 h post-infection with an MOI of approximately 5 is shown. Black columns correspond to uninfected cells, dark grey columns correspond to strain JF4278 (co-culture), while light grey columns correspond to strain L22/93 (co-culture). The data shown are the mean values of duplicates of three independent experiments. The x-axis represents the conditions tested (Bomac: Bomac cells free of BVDV; Bomac + BVDV: Bomac infected with BVDV), while y-axis represents the values of the measured test relative to untreated and uninfected cells. The values obtained for both Bomac cell lines infected with *M. bovis* were normalized to values of the corresponding uninfected and untreated cells of each cell line. Standard deviations of individual measurements are indicated as bars. *P < 0.05, **P < 0.01, ***P < 0.001.

**Figure 6:**
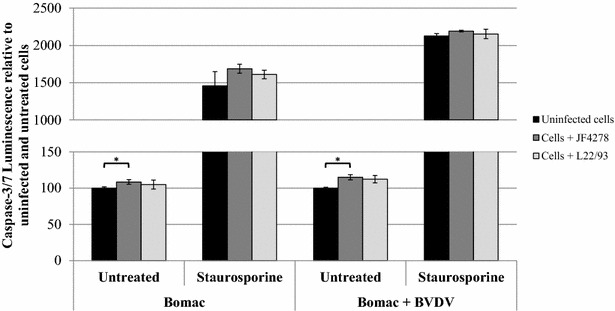
**Apoptosis assay.** Time point 24 h post-infection with an MOI of approximately 5 is shown. Black columns correspond to uninfected cells, dark grey columns correspond to strain JF4278 (co-culture), while light grey columns correspond to strain L22/93 (co-culture). The data shown are the mean values of duplicates of three independent experiments. The x-axis represents the conditions tested (Bomac: Bomac cells free of BVDV; Bomac + BVDV: Bomac infected with BVDV), while y-axis represents the values of the measured test relative to untreated and uninfected cells. The values obtained for both Bomac cell lines infected with *M. bovis* were normalized to values of the corresponding uninfected and untreated cells of each cell line. Standard deviations of individual measurements are indicated as bars. *P < 0.05, **P < 0.01, ***P < 0.001. In order to show the statistical differences observed for untreated cells, two graphs using different scales in the y-axis were merged.

In order to assess viability, cytotoxicity and induction of apoptosis in both Bomac cell lines, the ApoTox-Glo™ Triplex Assay was used. Cell viability was evaluated for both cell lines with or without infection with both strains of *M. bovis*. These Bomac cells were treated chemically with staurosporine or left untreated. No significant decrease in cell viability was observed after infection with strains L22/93 and JF4278 in both cell lines (Figure 4; untreated). As expected, staurosporine severely decreased cell viability in both cell lines (*P*-values: Bomac: 0.0001; Bomac with BVDV: 0.0031) (Figure 4; staurosporine). The cytotoxicity measurements showed a higher variability than the viability ones. Generally, infection with *M. bovis* seems to slightly decrease the measured cytotoxicity (Figure 5; untreated). However, the opposite is true in the case of strain JF4278 in Bomac cells free of BVDV (Figure 5: untreated). The cytotoxic effect measured after infection with the strain JF4278 in cells free of BVDV was confirmed by the ratio cytotoxicity-viability relative to *M. bovis* uninfected cells (Additional file 5). This was not observed in the case of Bomac cells infected with BVDV, although it has to be mentioned that for the untreated cell line, large variations were noted among the three different experiments. Treatment with staurosporine induced cytotoxicity in Bomac cells free of BVDV compared to untreated cells (*P*-value 0.0005). In Bomac cells infected with BVDV, an increase in cytotoxicity was not observed after treatment with staurosporine (*P*-value 0.1149), probably due to high variability in cytotoxicity measurements in untreated cells (Figure 5; staurosporine). Interestingly, there is a statistically significant reduction of cytotoxicity in Bomac cells free of BVDV after treatment with staurosporine and infection with *M. bovis* (Figure 5; staurosporine). Staurosporine strongly induced apoptosis in both Bomac cell lines (*P*-values: Bomac: 0.0065; Bomac with BVDV: < 0.0001) (Figure 6; staurosporine) and the values are confirmed by the ratios measuring apoptosis–viability relative to uninfected cells (Additional file 6). Cell infection with *M. bovis* strain JF4278 slightly induced apoptosis (Figure 6; untreated). This was also observed after induction of apoptosis with staurosporine but without statistical significance (Figure 6).

## Discussion

Observations performed on necropsy material suggested a potential intracellular stage of *M. bovis* [18–20, 35]. Recently, in vitro studies confirmed cell uptake of *M. bovis* and subsequent intracellular localization [21, 36, 37]. These studies were mainly carried out in primary bovine cells. Although primary cells have the advantage of being closer to the in vivo situation, cell lines are more convenient to work with, generally display less variability, and are amenable to genetic engineering. This is an obvious asset when dissecting the molecular and cellular mechanisms involved in host-*M. bovis* interactions that are not dependent on the variability of individual hosts [51]. Therefore, we chose a widely used bovine macrophage cell line (Bomac cells) for further studies. The model was kept as simple as possible. Opsonins or other serum components were not added and, thus, this model does not reflect the role of macrophages in vivo. Interestingly, the routine screening performed in our department to detect BVDV in cell cultures revealed a positive result for the Bomac cell line available in the laboratory. By curing the Bomac cell line of the contaminating BVDV type-2 (Marti and Schweizer, manuscript in preparation), two cell lines, i.e., Bomac cells with and without persisting BVDV, were made available and both cell lines showed no macroscopic differences. This has allowed the investigation of the potential synergism of BVDV and *M. bovis* as suggested in previous studies [31, 52, 53]. Furthermore, considering the switch in central Europe from subclinical to clinical mastitis in the mid-2000s [45], two different *M. bovis* strains were selected for the study. The two strains belong to two different clonal complexes with one strain being isolated before 2007 and one after 2007 in Switzerland when cases of severe mastitis started to be observed. The aim of this study was to correlate cell infection with cell viability, cytotoxicity and induction of apoptosis with the two strains of *M. bovis* belonging to two different CC and to investigate a potential influence of BVDV.

Our findings show that both *M. bovis* strains were able to enter in and grow in co-culture with Bomac cells either in single infection with *M. bovis* or in co-infection with BVDV (Figure 3). The rate of uptake of the two bacterial strains was similar for Bomac cells infected with BVDV or free of BVDV (Figure 3). Additionally, no statistical significant differences were observed in the number of mycoplasmas of both strains recovered 54 h post-infection in both cell lines after gentamicin treatment. Major differences between Bomac cells infected with BVDV and free of BVDV were not observed with respect to mycoplasmal uptake and number of recovered *M. bovis* during co-cultures for 54 h. It has to be noted that growth of *M. bovis* strain JF4278 was observed in spent MEM-Earle medium possibly due to substances secreted by Bomac cells or due to intracellular components Bomac cells released into the medium upon death of Bomac cells. These findings suggest that the Bomac cells can be used as a model to investigate the molecular mechanisms involved in cell uptake of *M. bovis* and co-culture even if infected with BVDV. Additional parameters could be tested in the future to investigate other aspects of co-infection between *M. bovis* and BVDV or even test multiple co-infections. However, it has to be emphasized that this is an in vitro model lacking the activity of complement or other serum components that could modify the interaction between macrophages and *M. bovis*.

Concerning the assays evaluating Bomac cells responses, cell viability was not drastically changed after infection with *M. bovis* (Figure 4). A slight increase in cytotoxicity was measured after infection of Bomac cells free of BVDV with the strain JF4278 (Figure 5). This observation cannot be confirmed in the case of Bomac cells infected with BVDV because of the high variability of cytotoxicity measured in Bomac cells infected with BVDV. However, staurosporine induced apoptosis in both cell lines with the cytotoxicity signal being lower after infection with both strains of *M. bovis* in Bomac cells free of BVDV. Cell infection with *M. bovis* strain JF4278 in Bomac cells with and without BVDV slightly induced apoptosis. This trend was also noticed after induction of apoptosis with staurosporine. Previous studies investigating apoptosis in monocytes/macrophages after infection with *M. bovis* showed a delayed apoptosis induced by this mycoplasmal species [21, 22, 37]. Delayed apoptosis could not be observed with the test used in this study. However, vanden Bush and Rosenbusch showed induction of apoptosis in lymphocytes after infection with *M. bovis* [23] but the cell response can be different between macrophages and lymphocytes. Interestingly, Zhang et al. recently showed induction of apoptosis in Bomac cells after incubation with a recombinant nuclease of *M. bovis* (MBOV_RS2825) associated with cytotoxicity [54].

In summary, no differences were observed at the level of cell uptake and growth in co-culture between the two strains of *M. bovis* and between cells infected or not with BVDV. Cell uptake and co-culture of *M. bovis* with both Bomac cell types did not decrease cell viability when cells were untreated with staurosporine. However, a slight increased cytotoxicity was observed after infection of Bomac cells without BVDV with the strain JF4278. Additionally, a small increase in apoptosis was measured after infection of both cell lines with *M. bovis* strain JF4278. These results show that the BVDV present in the infected cell line does not substantially change the parameters tested in this study. However, the BVDV status of in vitro models should be tested for research purposes especially considering that a wide variety of cells are known to be contaminated with BVDV [55–57]. Although cell cultures are generally tested to detect mycoplasma contaminations, examinations for BVDV are not widespread [58]. Although the curing of BVDV is time-consuming and might not always be successful, the information about its presence or absence is essential and researchers should at least test for it and be aware whether the virus is present or not because the virus might still influence other cellular parameters. Since many cells from cattle and other ruminants are infected with BVDV, this study aimed to measure different bovine cell responses between BVDV-free and BVDV-infected cells after *M. bovis* infection. This aspect is relevant not only for the interpretation of experimental data, but also because this virus was suggested to interact with *M. bovis* during natural infection.

## Additional files

**Additional file 1.** **Number of bacteria added per well and number of bacteria at time point 0 (after washing with PBS).** Black columns correspond to number of bacteria added per well. Grey columns correspond to the number of bacteria per well at time 0 after washing with PBS (no bovine cells). Horizontally striped columns correspond to the number of bacteria per well at time 0 after washing with PBS (with bovine cells). The x-axis represents the conditions tested (Bomac: Bomac cells free of BVDV; Bomac + BVDV: Bomac infected with BVDV), while y-axis represents the log10 CFU/well. The data shown are the mean values of triplicates of three independent experiments. Standard deviations of individual measurements are indicated as vertical bars. *P < 0.05, **P < 0.01, ***P < 0.001.

**Additional file 2.**** Staurosporine concentrations tested in preliminary assay.** Time point 6 h post-treatment of bovine cells with different concentrations of staurosporine. The x-axis represents the cells tested (Bomac: Bomac cells free of BVDV; Bomac + BVDV: Bomac infected with BVDV) with different concentrations of staurosporine, while y-axis represents the values of the measured test relative to untreated and uninfected cells.

**Additional file 3.** **Survival of** ***M. bovis*** **strain JF4278 in MEM-Earle medium and in spent MEM-Earle medium (medium incubated with Bomac cells for 24 and 48** **h).** The dotted line represents results with fresh MEM-Earle. The straight lines represent results with spent medium of Bomac cells, while the dashed lines represent results with spent medium of Bomac cells infected with BVDV. The x-axis indicates the timepoints and the y-axis the log10 CFU/mL. The data shown are the mean values of three independent experiments. Standard deviations of individual measurements per time point are indicated as vertical bars.

**Additional file 4.** **Survival of** ***M. bovis*** **strain L22/93 in MEM-Earle medium and in spent MEM-Earle medium (medium incubated with Bomac cells for 24 and 48** **h).** The dotted line represents results with fresh MEM-Earle. The straight lines represent results with spent medium of Bomac cells, while the dashed lines represent results with spent medium of Bomac cells infected with BVDV. The x-axis indicates the timepoints and the y-axis the log10 CFU/mL. The data shown are the mean values of three independent experiments. Standard deviations of individual measurements per time point are indicated as vertical bars.

**Additional file 5.** **Ratio of cytotoxicity/viability relative to uninfected cells.** Confirmation of the cytotoxic effect of staurosporine treatment and *M. bovis* infection. To account for the strongly reduced viability measures in staurosporine treated samples, the ratio of cytotoxicity signals and viability signals relative to uninfected and untreated cells are shown in the table.

**Additional file 6.** **Ratio of apoptosis/viability relative to uninfected cells.** Confirmation of the apoptosis induction after staurosporine treatment and *M. bovis* infection. To account for the strongly reduced viability measures in staurosporine treated samples, the ratio of apoptosis signals and viability signals relative to uninfected and untreated cells are shown in the table.
