# Supplementary figures and images for: Mycoplasma bovis co-infection with bovine viral diarrhea virus in bovine macrophages
Source: Vet Res. 2018 Jan 9;49:2. doi: 10.1186/s13567-017-0499-1 (PMC5761114; doi:10.1186/s13567-017-0499-1)

## Slide 1
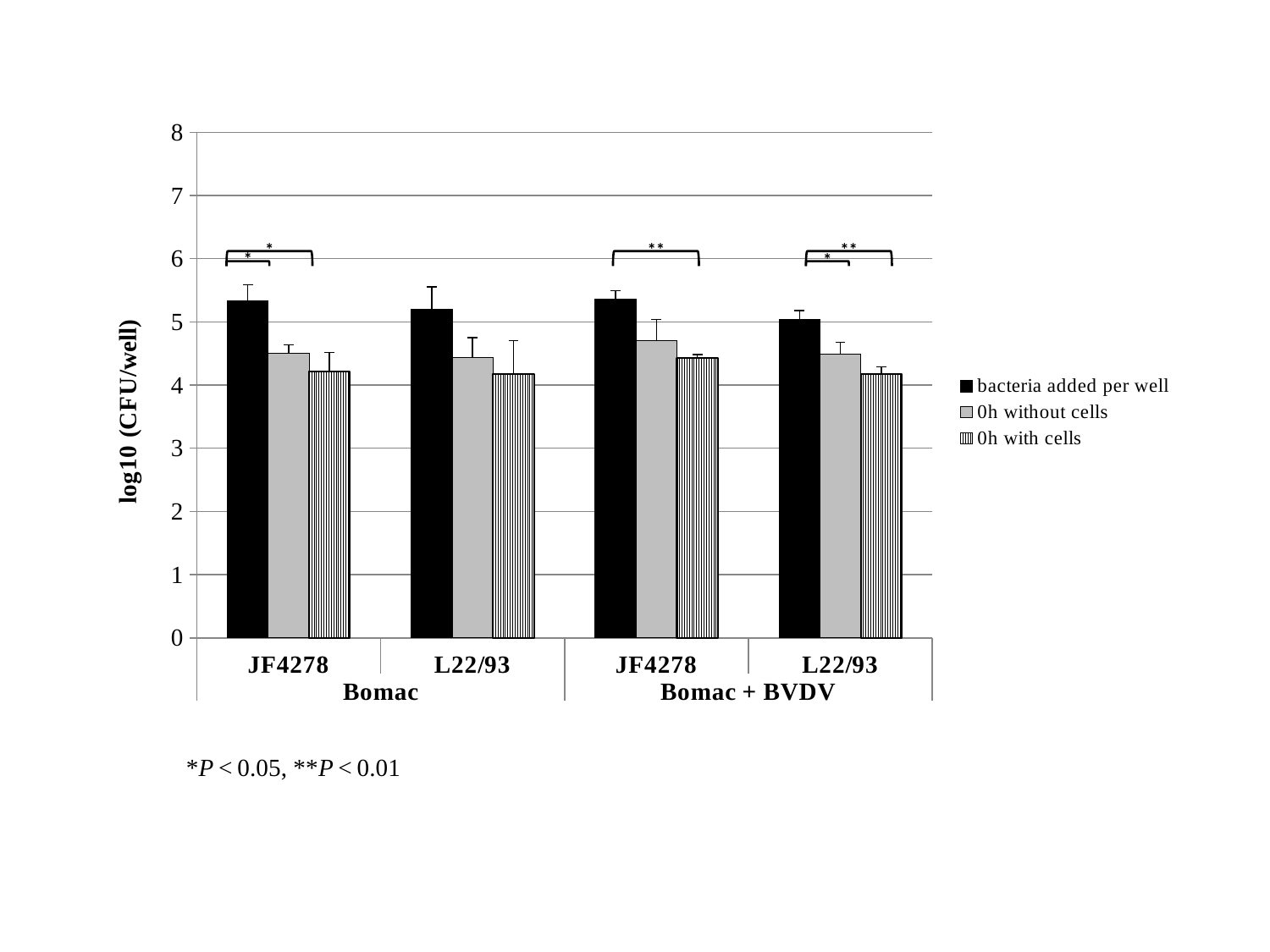

### Chart
| Category | bacteria added per well | 0h without cells | 0h with cells |
|---|---|---|---|
| JF4278 | 5.335861340673006 | 4.502911621111108 | 4.219864914444444 |
| L22/93 | 5.199605808867767 | 4.43952671444445 | 4.172927409999994 |
| JF4278 | 5.357901872889381 | 4.697709197777777 | 4.425457906666667 |
| L22/93 | 5.03229318742787 | 4.495234208888887 | 4.175250926666666 |
**
**
*
*
*
*P < 0.05, **P < 0.01

Supplement: Supplementary file 1 — Additional file 1. Number of bacteria added per well and number of bacteria at time point 0 (after washing with PBS). Black columns correspond to number of bacteria added per well. Grey columns correspond to the number of bacteria per well at time 0 after washing with PBS (no bovine cells). Horizontally striped columns correspond to the number of bacteria per well at time 0 after washing with PBS (with bovine cells). The x-axis represents the conditions tested (Bomac: Bomac cells free of BVDV; Bomac + BVDV: Bomac infected with BVDV), while y-axis represents the log10 CFU/well. The data shown are the mean values of triplicates of three independent experiments. Standard deviations of individual measurements are indicated as vertical bars. *P < 0.05, **P < 0.01, ***P < 0.001. [file 13567_2017_499_MOESM1_ESM.pptx]

## Slide 1
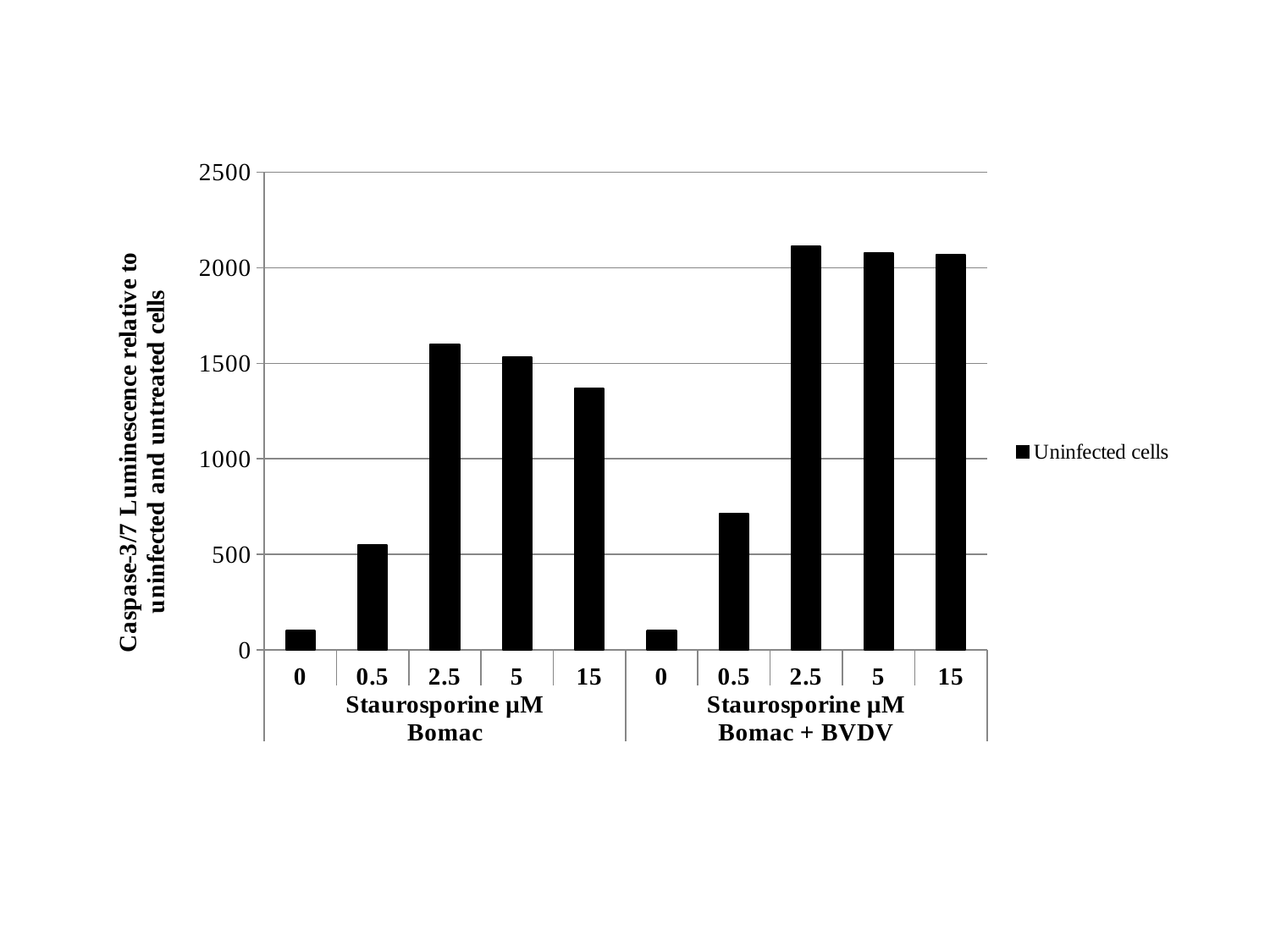

### Chart
| Category | Uninfected cells |
|---|---|
| 0 | 100.0 |
| 0.5 | 548.3554933519944 |
| 2.5 | 1597.900629811056 |
| 5 | 1533.3100069979 |
| 15 | 1370.888733379986 |
| 0 | 100.0 |
| 0.5 | 711.6427432216906 |
| 2.5 | 2111.363636363636 |
| 5 | 2076.475279106858 |
| 15 | 2067.224880382768 |

Supplement: Supplementary file 2 — Additional file 2. Staurosporine concentrations tested in preliminary assay. Time point 6 h post-treatment of bovine cells with different concentrations of staurosporine. The x-axis represents the cells tested (Bomac: Bomac cells free of BVDV; Bomac + BVDV: Bomac infected with BVDV) with different concentrations of staurosporine, while y-axis represents the values of the measured test relative to untreated and uninfected cells. [file 13567_2017_499_MOESM2_ESM.pptx]
