# Supplementary material for: Mycoplasma bovis co-infection with bovine viral diarrhea virus in bovine macrophages
Source: Vet Res. 2018 Jan 9;49:2. doi: 10.1186/s13567-017-0499-1 (PMC5761114; doi:10.1186/s13567-017-0499-1)
Supplement: Supplementary file 3 — Additional file 3. Survival of M. bovis strain JF4278 in MEM-Earle medium and in spent MEM-Earle medium (medium incubated with Bomac cells for 24 and 48 h). The dotted line represents results with fresh MEM-Earle. The straight lines represent results with spent medium of Bomac cells, while the dashed lines represent results with spent medium of Bomac cells infected with BVDV. The x-axis indicates the timepoints and the y-axis the log10 CFU/mL. The data shown are the mean values of three independent experiments. Standard deviations of individual measurements per time point are indicated as vertical bars. [file 13567_2017_499_MOESM3_ESM.pptx]

## Slide 1
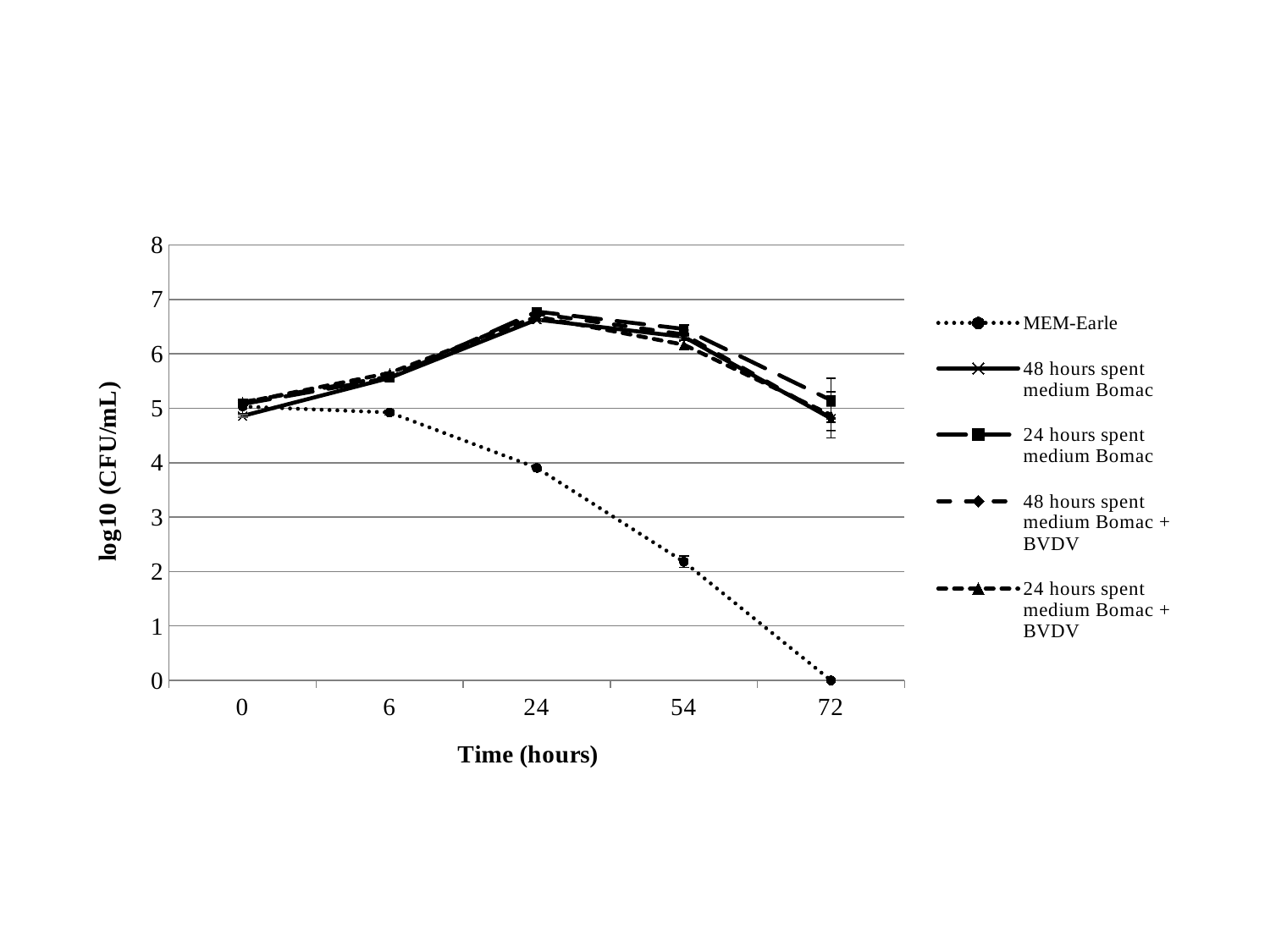

### Chart
| Category | MEM-Earle | 48 hours spent medium Bomac | 24 hours spent medium Bomac | 48 hours spent medium Bomac + BVDV | 24 hours spent medium Bomac + BVDV |
|---|---|---|---|---|---|
| 0 | 5.030983056388616 | 4.858625592719427 | 5.077068771647 | 5.112612733832506 | 5.102033712706315 |
| 6 | 4.922182628454148 | 5.557453616480945 | 5.55955744134477 | 5.58329837346521 | 5.648653881275255 |
| 24 | 3.904920028767045 | 6.628714249055389 | 6.774965713414581 | 6.739310600512077 | 6.682376077276687 |
| 54 | 2.178606113690518 | 6.311719342739269 | 6.457021681513496 | 6.35519383510392 | 6.166505408104197 |
| 72 | 0.0 | 4.811967504289282 | 5.14736113328843 | 4.821835387082896 | 4.876811245820735 |
