# Supplementary material for: Mycoplasma bovis co-infection with bovine viral diarrhea virus in bovine macrophages
Source: Vet Res. 2018 Jan 9;49:2. doi: 10.1186/s13567-017-0499-1 (PMC5761114; doi:10.1186/s13567-017-0499-1)
Supplement: Supplementary file 4 — Additional file 4. Survival of M. bovis strain L22/93 in MEM-Earle medium and in spent MEM-Earle medium (medium incubated with Bomac cells for 24 and 48 h). The dotted line represents results with fresh MEM-Earle. The straight lines represent results with spent medium of Bomac cells, while the dashed lines represent results with spent medium of Bomac cells infected with BVDV. The x-axis indicates the timepoints and the y-axis the log10 CFU/mL. The data shown are the mean values of three independent experiments. Standard deviations of individual measurements per time point are indicated as vertical bars. [file 13567_2017_499_MOESM4_ESM.pptx]

## Slide 1
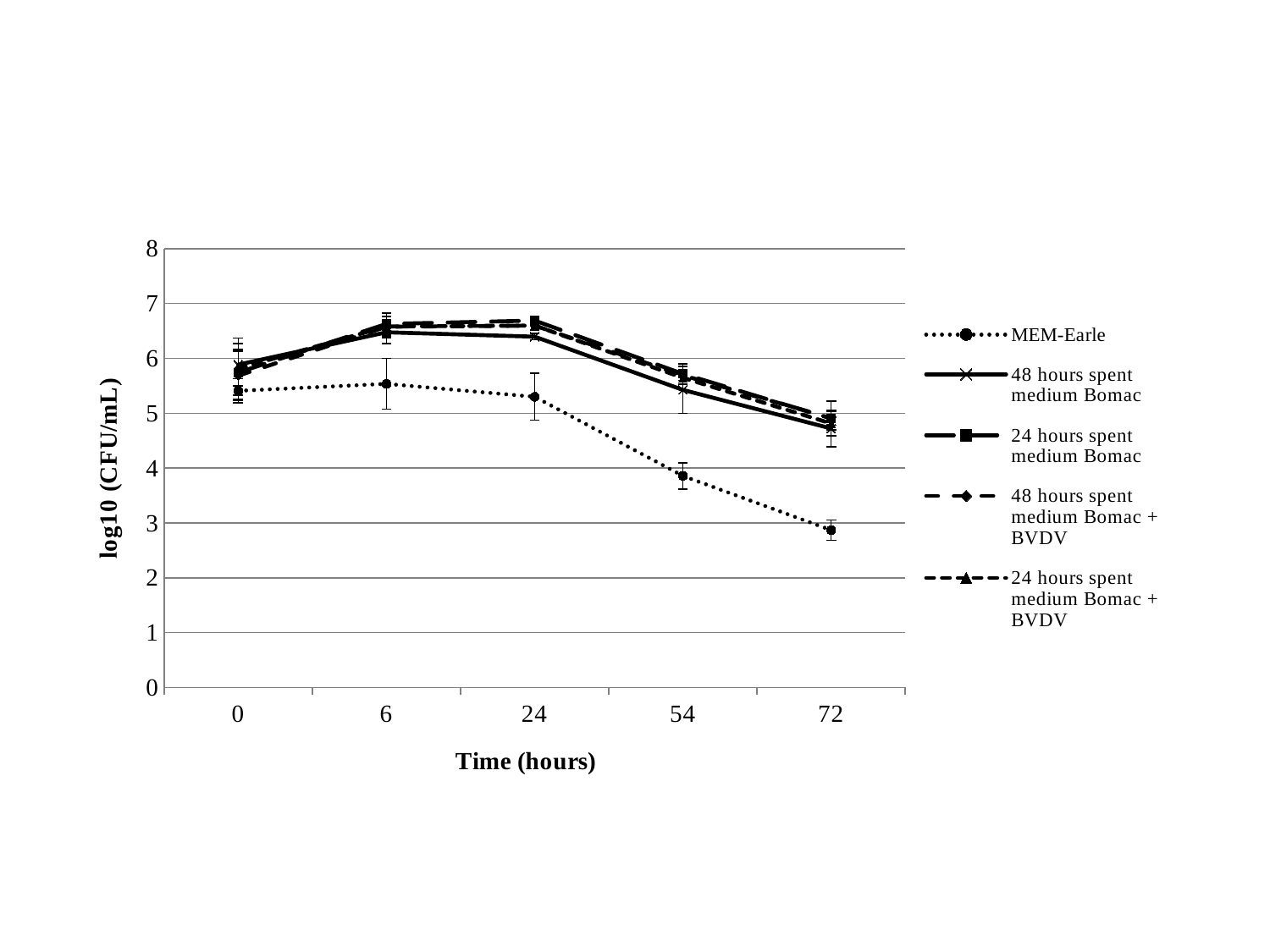

### Chart
| Category | MEM-Earle | 48 hours spent medium Bomac | 24 hours spent medium Bomac | 48 hours spent medium Bomac + BVDV | 24 hours spent medium Bomac + BVDV |
|---|---|---|---|---|---|
| 0 | 5.40746932544723 | 5.884833928359868 | 5.741954081137332 | 5.683672727381244 | 5.811705377384746 |
| 6 | 5.5346693088742 | 6.474551768119436 | 6.627059659751375 | 6.576650636746881 | 6.581108998704532 |
| 24 | 5.298622805047706 | 6.394909748354466 | 6.688799091430437 | 6.594840387630446 | 6.597211144136788 |
| 54 | 3.857054616409163 | 5.424954794578332 | 5.717245938488678 | 5.691334267640776 | 5.645416371387836 |
| 72 | 2.868912788063383 | 4.721585937968424 | 4.904734738706979 | 4.90900937579356 | 4.812178445391822 |
