# Supplementary material for: Mycoplasma bovis co-infection with bovine viral diarrhea virus in bovine macrophages
Source: Vet Res. 2018 Jan 9;49:2. doi: 10.1186/s13567-017-0499-1 (PMC5761114; doi:10.1186/s13567-017-0499-1)
Supplement: Supplementary file 5 — Additional file 5. Ratio of cytotoxicity/viability relative to uninfected cells. Confirmation of the cytotoxic effect of staurosporine treatment and M. bovis infection. To account for the strongly reduced viability measures in staurosporine treated samples, the ratio of cytotoxicity signals and viability signals relative to uninfected and untreated cells are shown in the table. [file 13567_2017_499_MOESM5_ESM.docx]

| **Cell treatment** | **Bomac type** | **Uninfected cells (SD^1^)** | **Cells+JF4278 (SD^1^)** | **Cells+L22/93 (SD^1^)** |
| --- | --- | --- | --- | --- |
| **Untreated** | No BVD virus | 100 (±11.80) | 169.67 (±21.11) | 83.31 (±10.22) |
|  | BVD virus | 100 (±53.08) | 80.21 (±17.01) | 40.44 (±0.87) |
| **Staurosporine** | No BVD virus | 1068.51 (±14.28) | 477.98 (±35.29) | 771.10 (±14.36) |
|  | BVD virus | 340.58 (±15.14) | 309.61 (±20.68) | 287.90 (±67.21) |

^1^SD: standard deviation
