# Supplementary material for: Mycoplasma bovis co-infection with bovine viral diarrhea virus in bovine macrophages
Source: Vet Res. 2018 Jan 9;49:2. doi: 10.1186/s13567-017-0499-1 (PMC5761114; doi:10.1186/s13567-017-0499-1)
Supplement: Supplementary file 6 — Additional file 6. Ratio of apoptosis/viability relative to uninfected cells. Confirmation of the apoptosis induction after staurosporine treatment and M. bovis infection. To account for the strongly reduced viability measures in staurosporine treated samples, the ratio of apoptosis signals and viability signals relative to uninfected and untreated cells are shown in the table. [file 13567_2017_499_MOESM6_ESM.docx]

| **Cell treatment** | **Bomac type** | **Uninfected cells (SD^1^)** | **Cells+JF4278 (SD^1^)** | **Cells+L22/93 (SD^1^)** |
| --- | --- | --- | --- | --- |
| **Untreated** | No BVD virus | 100 (±1.88) | 117.27 (±3.42) | 113.11 (±6.66) |
|  | BVD virus | 100 (±1.27) | 119.93 (±3.79) | 105.58 (±6.66) |
| **Staurosporine** | No BVD virus | 3484.463 (±455.46) | 3981.83 (±142.71) | 5073.66 (±181.36) |
|  | BVD virus | 3950.16 (±60.49) | 4050.59 (±19.39) | 4047.28 (±119.87) |

^1^SD: standard deviation
